# Chemical modification allows phallotoxins and amatoxins to be used as tools in cell biology

**DOI:** 10.3762/bjoc.8.233

**Published:** 2012-11-27

**Authors:** Jan Anderl, Hartmut Echner, Heinz Faulstich

**Affiliations:** 1 Heidelberg Pharma GmbH, Schriesheimer Str. 101, 68526 Ladenburg, Germany; 2 University of Tübingen, Medical School, Hoppe-Seyler-Str. 3, 72076 Tübingen, Germany,; 3 Max-Planck Institute for Medical Research, Jahnstr. 29, 69120 Heidelberg, Germany

**Keywords:** amatoxins, cellular uptake, endocytosis, peptides, phalloidin, phallotoxins

## Abstract

Phallotoxins inhibit the dynamics of microfilaments in cells and lead to apoptosis. Due to poor cellular uptake these effects cannot be studied in live cells, even at millimolar toxin concentrations, nor can phalloidin be used for the elimination of tumor cells. Uptake is greatly enhanced by conjugation of phallotoxins to either lipophilic or polycationic moieties, such as oleic acid, polylysine, or Tat-peptide. These conjugates were lethally toxic for cells, e.g., mouse fibroblasts or Jurkat leukemia cells, in the micromolar range. Uptake into cells starts with the attachment of the toxin conjugates to the plasma membrane, followed by endocytosis and, in most cases, cleavage of the toxin from the carrier. Interestingly, the internalization rate of phalloidin into cells was also significantly increased by the fluorescent moiety tetramethylrhodaminyl, as well as by high molecular weight methoxy-polyethyleneglycol, two compounds unknown so far for their uptake-mediating activity. Conjugation to carriers as investigated in this work will allow the use of phallotoxins in experimental cell biology and possibly in tumor therapy. The findings obtained with phallotoxins could be applied also to the family of amatoxins, where α-amanitin, for example, when conjugated to oleic acid was more than 100-fold more toxic for cells than the native toxin. This suggests the possibility of a more general use of the moieties examined here to enhance the uptake of hydrophilic peptides, or drugs, into live cells.

## Introduction

Phallotoxins and amatoxins, the two families of toxic cyclopeptides produced by the green death cap *Amanita phalloides*, have been the subject of intense biochemical research for decades [1]. Although produced by the same mushroom and of similar structure, the two peptide families have totally different cellular targets. Phallotoxins, such as phalloidin, bind to polymeric actin, thus stabilizing microfilaments and decreasing the amount of monomeric actin in equilibrium with the filaments. This interaction is in the nanomolar range and highly specific: no other targets for phalloidin in the cell are known. Amatoxins, such as the main toxin α-amanitin, bind to RNA-polymerases II of eukaryotic cells, thus inhibiting the transcription process at nanomolar concentrations. Also this interaction is specific, since RNA-polymerases I are not inhibited at all, whereas RNA-polymerases III are inhibited at amanitin concentrations ca. 1000 times higher than for RNA-polymerases II [2–3]. The fact that both the cytoskeleton and the eukaryotic transcription machine are complex structures and still under investigation, may explain the continuing interest in these two kinds of specific inhibitors.

Phalloidin has been used to study actin dynamics in vitro [1], and in microscopic studies after microinjection into single cells [4]. Beside such experiments phalloidin conjugated to fluorescent moieties is widely used for visualizing filamentous actin in fixed cells [5–6]. In all these applications, cell-free systems were used, allowing direct access of phallodin to its target. Similarly, α-amanitin was mainly used with isolated nuclei or the solubilized enzyme, as recently reported for stabilizing yeast RNA-polymerase II in an X-ray analysis [7].

For both phallotoxins and amatoxins, experience with live cells is limited by the fact that the peptides cross the plasma membrane barrier only very slowly. Poor uptake rates of phallotoxins and amatoxins have been observed for most mammalian cells. Mammalian hepatocytes are an exception: they display transporting proteins on their sinusoidal surface (such as OATP1B1 and OATP1B3 of human hepatocytes [8–10]), which internalize phallotoxins and amatoxins. It is through the presence of the transporting protein OATP1B3 on human hepatocytes, for example, that amatoxins are feared as liver toxins causing the majority of fatal human mushroom poisonings worldwide.

The aim of this study was to explore known lipophilic and polycationic internalization-mediating moieties for their applicability with phallotoxins and amatoxins, and to find novel internalization-mediating moieties and investigate the potential of their conjugates with toxins as specific inhibitors in cultured cells. The internalization-mediating moieties used in this study were either lipophilic in nature (such as oleic acid), or multicationic (such as polylysine and octarginine): two features applying also to so-called membrane-transducing peptides [11–12].

## Results

### Preparation of phallotoxin derivatives

**Figure 1:**
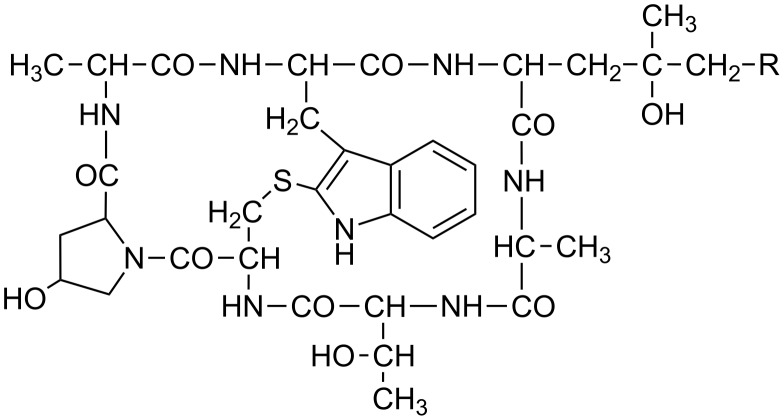
Chemical structure of phalloidin with the attachment site (R) used for conjugation to uptake-mediating moieties.

Attachment sites in phalloidin (Figure 1) for conjugation with uptake-mediating moieties were chosen based on our knowledge of structure–activity relationships in phalloidin [1]: As the side chains in the small heterodet peptide ring are known to be involved in actin binding they were not used for derivatization. In the larger peptide ring, on the other hand, the dihydroxylated leucine moiety is juxtaposed to the actin binding site and thus appeared as most promising for derivatization. Accordingly, all residues investigated in this study were attached to the Cδ-atom of the dihydroxylated leucine moiety, either as esters or amides (Table 1). Large residues, which might disturb the interaction of a phalloidin derivative with actin through steric hindrance, were coupled via disulfide-containing linkers, which would be reduced inside the cell so as to release a defined thiol derivative of phalloidin (Table 1).

**Table 1:** Structures of phalloidin derivatives, their relative affinity values for α-actin as compared to phalloidin and their IC_50_ values of cell growth inhibition after incubation for 72 h as determined by MTT cell proliferation assay (n.a.: not assayed).

	R	Name/designation	Relative affinity to α-actin [%]	IC_50_ values NIH 3T3 fibroblasts [µM]	IC_50_ values Jurkat cells [µM]

**1**	OH	phalloidin	100	ca. 1,000	ca. 1,000
**1a**	OCOC_6_H_5_	(**1**)-benzoate	76	92	94
**1b**	OCOC_6_H_4_*o*-OH	(**1**)-salicylate	81	364	96
**1c**	OCOC_7_H_15_	(**1**)-octanoate	12	44	37
**1d**	OCOC_13_H_27_	(**1**)-myristate	7	11	9
**1e**	OCO(CH_2_)_7_CHCH(CH_2_)_7_CH_3_	(**1**)-oleate	4	3	2
**2**	NH_2_	aminophalloidin	2	ca. 1,000	ca. 1,000
**2a**	NHCOC_6_H_5_	*N*-benzoyl-(**2**)	74	693	599
**2b**	NHCO(CH_2_)_7_CHCH(CH_2_)_7_CH_3_	*N*-oleoyl-(**2**)	5	883	630
**2c**	NHCO(CH_2_)_2_SS(Ac)CysGlyTyrGly- -Arg(Lys)_2_(Arg)_2_Glu(Arg)_3_OH	(**2**)-Tat-peptide	3	4	3
**2d**	NHCO(CH_2_)_2_SS(Ac)CysGly(Arg)_8_OH	(**2**)-octarginine	2	11	7
**2e**	NHCO(CH_2_)_6_CONH(Lys)_210_	(**2**)-poly-(L)-lysine_28,000_	40	5	4
		(**2**)-poly-(D)-lysine_28,000_	38	54	37
**2f**	NHCO(CH_2_)_2_SS(CH_2_)_2_CONH(Lys)_210_	(**2**)-(SS) poly-(L)-lysine_28,000_	39	3	2
		(**2**)-(SS) poly-(D)-lysine_28,000_	39	4	2
**2g**	NH(CH_2_)_2_SS(CH_2_)_2_CONH(PEG)_800_	(**2**)-(SS) PEG_800_	8	167	98
**2h**	NH(CH_2_)_2_SS(CH_2_)_2_CONH(PEG)_5.200_	(**2**)-(SS) PEG_5,200_	4	77	50
**2i**	NH(CH_2_)_2_SS(CH_2_)_2_CONH(PEG)_23.000_	(**2**)-(SS) PEG_23,000_	1	10	10
**2f +DTT**	NH(CH_2_)_2_SH	*N*-(2-mercaptoethyl)-(**2**)-SH	37	n.a.	n.a.
**3**	for structure see Figure 6a	dithiolanoaminophalloidin TRITC labeled	25	11	n.a

### Actin binding

All phalloidin derivatives were tested for their affinity to muscle actin (α-actin), which is used as a model for β-actin present in nonmuscle cells. This parameter was important, since cytotoxicity depends not only on membrane permeability but also on actin affinity. Moreover, this parameter will hint on whether phalloidin was cleaved from its carrier inside the cell, in cases where low actin affinity of a phalloidin derivative was combined with high cytotoxicity. Relative affinity values of the phalloidin derivatives to muscle actin are shown in Table 1.

#### Growth inhibition of mouse fibroblasts

**Figure 2:**
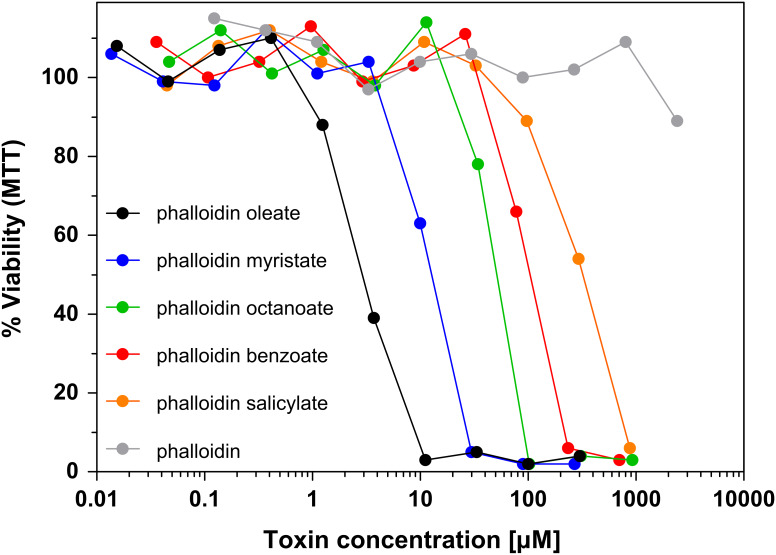
Cytotoxicity of phalloidin derivatives. NIH 3T3 mouse fibroblasts were incubated with various concentrations of phalloidin and phalloidin derivatives. Cell viability was determined after 72 h incubation time by MTT assay.

**Figure 3:**
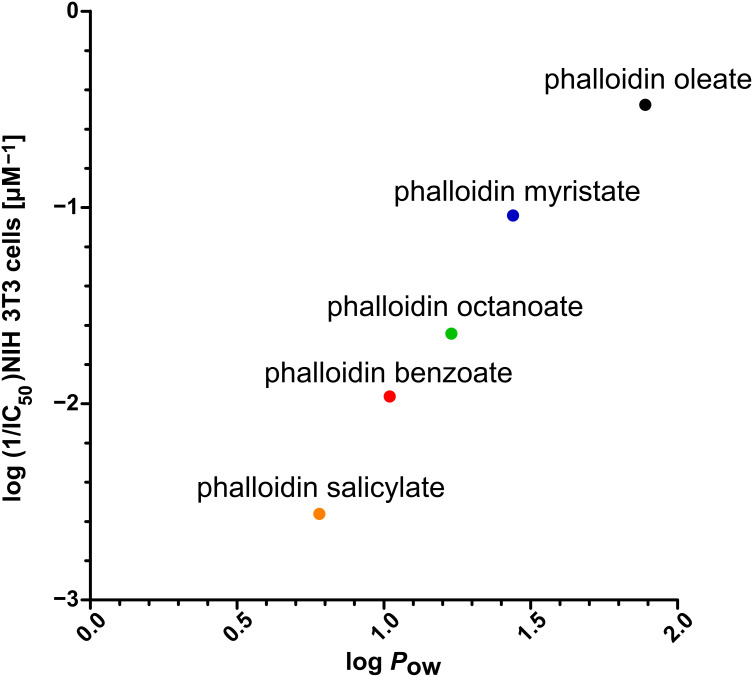
*n*-Octanol/water distribution coefficients (log *P*_ow_) of the hydrophobic phalloidin derivatives of Figure 2 plotted versus cell toxicity (log (1/IC_50_) in NIH 3T3 mouse fibroblasts.

By using the MTT cell proliferation assay, each phalloidin conjugate was examined for its capacity to inhibit the growth of mouse fibroblasts in vitro after 72 h incubation time (Table 1). Phalloidin displayed no antiproliferative activity up to a concentration of 10^−3^ M in the culture medium. In contrast, the most lipophilic ester derivative, phalloidin oleate (**1e**), showed an IC_50_ value of proliferation inhibition of 2.5 × 10^−6^ M, and was thus ca. 1000 times more active than phalloidin. Other esters (**1a**–**1d**) exhibited increasing cytotoxic activities with increasing hydrophobicity (Figure 2). In order to examine a possible relationship between cytotoxicity and hydrophobicity we determined the octanol/water equilibrium distribution coefficient of the ester derivatives (log *P*_ow_ values) and found a linear relationship between log *P*_ow_ and IC_50_ values of cellular cytotoxicity (Figure 3). The ester derivatives of phalloidin are probably hydrolyzed inside the cell, e.g., by esterases or proteases, as suggested by the fact that the ester **1e** and the corresponding amide **2b** possess comparable affinities to actin (Table 1), but differ in their IC_50_ values by a factor of ca. 300. The effect is presumably a consequence of the faster hydrolysis of esters over amides by intracellular hydrolases.

**Figure 4:**
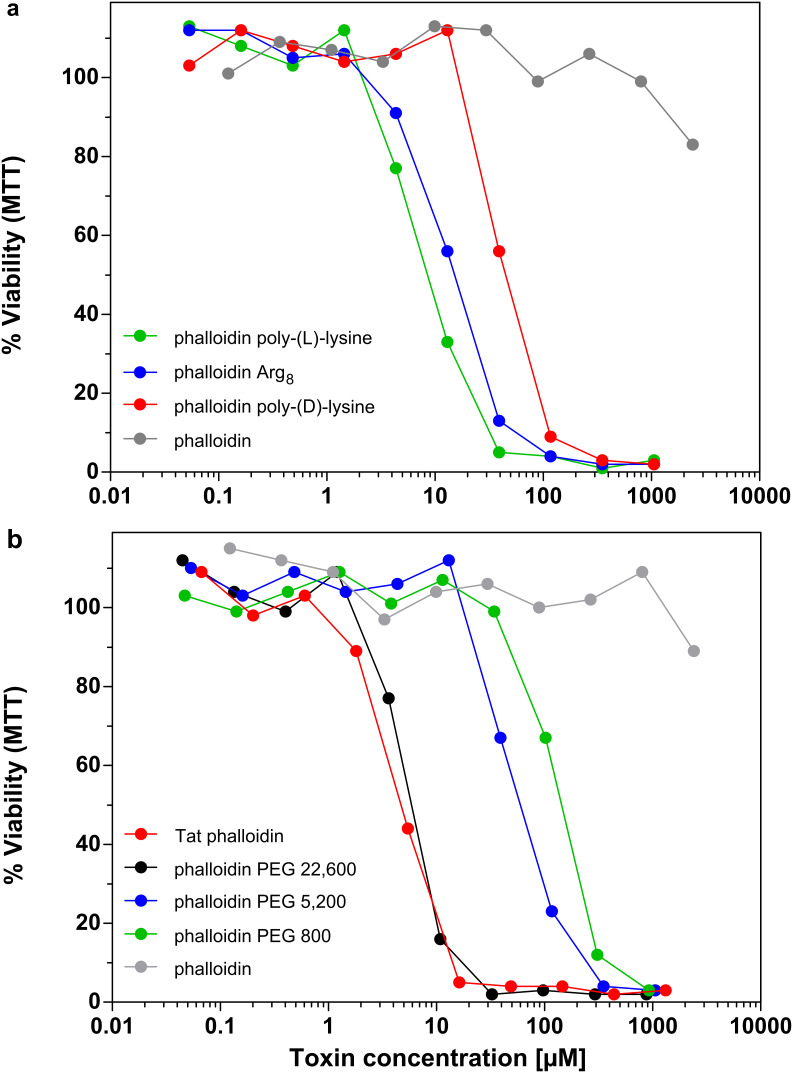
(a) and (b): NIH 3T3 mouse fibroblasts were incubated with various concentrations of phalloidin and phalloidin derivatives. Cell viability was determined after 72 h incubation time by MTT assay.

Polycationic derivatives of phalloidin, such as the polylysine conjugates, were highly toxic for mouse fibroblasts, and their antiproliferative activity was comparable to the most lipophilic derivative, phalloidin oleate (Figure 4a). Their toxicity was found to be dependent on the configuration of the polymer, since phalloidin bound to D-configurated polylysine was about 10 times less toxic than when bound to poly-(L)-lysine. This suggests that the release of a toxic phalloidin species inside the cell includes the enzymatic breakdown of the carrier. In agreement with this we found no difference between the L-configurated and the D-configurated carrier when the linker contained a disulfide bridge, arguing for the presence of a disulfide-reducing compartment inside the cells.

Likewise, high cytotoxicity was found for the octarginine conjugate (Figure 4a). From the fact that the cytotoxic activities of the polylysine derivative (ca. 150 residues) and the oligoarginine derivative (8 residues) are comparable, we argue that arginine residues are more effective in mediating internalization of phalloidin than are lysine residues.

For phalloidin bound to methoxy-polyethylene-glycol we found that cytotoxicity strictly depended on the molecular weight of the polymer chain (Figure 4b). Most active was the conjugate with the longest chain (*M*_r_ = 22,600). Cytotoxicity fell strikingly when the polymer chain was shortened (Table 1).

Coupling to Tat-peptide was also very effective in enhancing the cytotoxicity of phalloidin (Figure 4b), while the phalloidin conjugate with the Kaposi protein fragment (likewise claimed to enhance membrane permeability) was much less effective (data not shown).

Beside mouse fibroblasts, we investigated the phalloidin derivatives in several human leukemia and lymphoma cell lines, in order to find possible specificities for one or the other kind of tumor cell. However, Jurkat cells (Table 1) and all other cell lines tested (K562 cells, HL-60 cells, and Daudi cells) showed sensitivities comparable to mouse fibroblasts (see Supporting Information File 1).

#### Uptake kinetics

**Figure 5:**
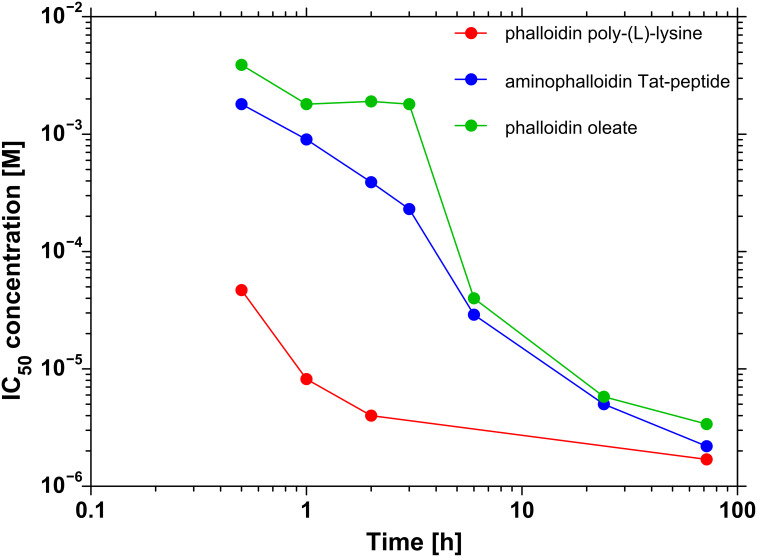
Time course of uptake in NIH 3T3 cells of three phalloidin derivatives as measured by the cytotoxicity after 72 h incubation during which the toxins were washed off after various exposure times.

Since the toxic effects of phalloidin develop slowly, growth inhibition was measured only after 72 h. During this period several partial processes must occur such as binding to the plasma membrane, internalization, processing and toxin release, etc., which cannot be distinguished. However, replacement of the toxin medium by toxin-free medium after various times of incubation would provide information on the time required for each of the toxin derivatives to bind to the cell surface. We compared the three most effective phalloidin conjugates, phalloidin oleate (**1e**), phalloidin-Tat conjugate (**2c**), and phalloidin polylysine (**2e**) (Figure 5) and found that the exposure times necessary to achieve, e.g., a 50% growth inhibition by a given toxin concentration after 72 h indeed varied considerably, from 2 h to 24 h. The results show that the polycationic derivative was bound much more rapidly than the lipophilic conjugate.

#### A fluorescent residue that enhances uptake of phalloidin into cells

**Figure 6:**
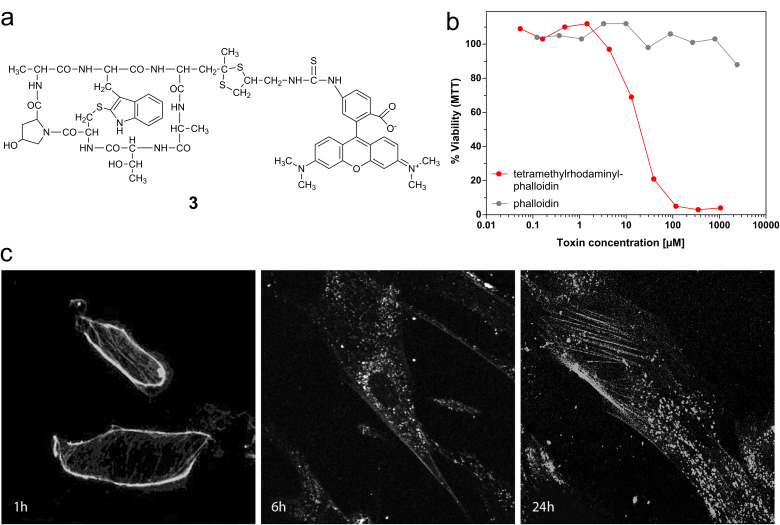
(a) Chemical structure of tetramethylrhodaminyl-phalloidin (**3**). (b) Growth inhibition of NIH 3T3 mouse fibroblasts by tetramethylrhodaminyl-phalloidin (**3**) versus phalloidin (**1**) after 72 h incubation time. (c) Binding and uptake of tetramethylrhodaminyl-phalloidin (**3**) in NIH 3T3 mouse fibroblasts after 1, 6 and 24 h, as documented by fluorescence microscopy.

Tetramethylrhodaminyl-phalloidin (Figure 6a) has been used to visualize actin fibers in fixed cells for 30 years. Here we show that the rhodamine residue also strongly enhanced cellular uptake, making this phalloidin derivative a tool for cell biology. With an IC_50_ value of 11 µM its toxicity is comparable to those of the most toxic phalloidin derivatives, phalloidin oleate (**1e**) and phalloidin-poly-(L)-lysine (**2e**). More importantly, rhodaminyl-phalloidin seems not to be cleaved inside the cell and, through its fluorescence, can report on the structure of its target protein, the actin filaments, albeit under toxic conditions.

Using rhodamine-labeled phalloidin, we could also study how membrane-permeable peptides are incorporated into the cell. Immediately after exposure the toxin was located on the plasma membrane of the cells as shown by fluorescence microscopy (Figure 6c), while after 6 h increasing amounts of the toxin were found in endocytotic vesicles. After 24 h most of the rhodamine-labeled toxin was still in endosomes, while some of it had found its target, as concluded from the decoration of filaments.

#### Phalloidin causes apoptosis of cells

Under the microscope, cells treated with membrane-permeable phalloidin derivatives appeared shrunken and developed blebs, as described for cells undergoing apoptosis. Treatment with annexin followed by flow cytometric analysis showed a fluorescence distribution typical for apoptosis and similar to that induced by camptothecin. Cells treated with native phalloidin were indistinguishable from controls [13].

#### Growth inhibition by amatoxin derivatives

Unlike the phallotoxins, the natural amatoxins are toxic in cell cultures, exhibiting IC_50_ values around 10^−6^ M. As amatoxins are more hydrophilic than phallotoxins, it seems unlikely that their membrane permeation capacity is larger than that of phallotoxins. The more likely explanation is that for amatoxins the threshold concentration lethal for cells is much lower than for phallotoxins (see Discussion).

**Figure 7:**
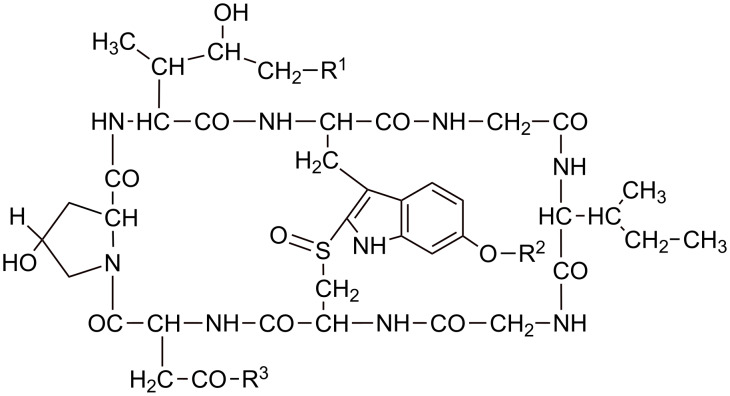
Chemical structure of amanitin with attachment site for conjugation to internalization-mediating moieties (R^1^, R^3^).

Two of the internalization-mediating residues investigated in the phallotoxin series, oleic acid and polylysine, were also tested for their uptake capacity in the amatoxin series. Structure–activity studies had shown that the primary OH group of the dihydroxy-isoleucine moiety (Figure 7) is not involved in RNA polymerase II binding and, hence, may be used for derivatization with oleic acid chloride (Table 2). In order to avoid concurrent acylation of the 6’-OH of tryptophan, the phenolic OH was methylated before acetylation. For coupling to polylysine the natural carboxy group of aspartic acid as present in β-amanitin was used, which after activation as *N*-hydroxysuccinimide ester reacted with ε-amino groups in polylysine. Since the polymeric carrier may be broken down inside the cell by proteases, we investigated both poly-(L)-lysine and poly-(D)-lysine as carriers, in order to study whether the biological availability of the amatoxin depended on the configuration of lysine in the polymeric carrier.

**Table 2:** Structures of amanitin derivatives and IC_50_ concentrations of growth inhibition in mouse fibroblasts after 72 h incubation time (MTT cell proliferation assay).

	R^1^	R^2^	R^3^		IC_50_ values NIH 3T3 fibroblasts [nM]

**4**	OH	OH	NH_2_	α-amanitin	6,200
**4a**	OCO(CH_2_)_7_CHCH(CH_2_)_7_CH_3_	OCH_3_	NH_2_	α-amanitin oleate	39
**5**	OH	OH	OH	β-amanitin	1,000
**5a**	OH	OH	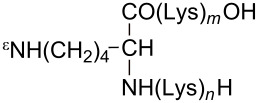	β-amanitin poly-(L)-lysine	10
**5b**	OH			β-amanitin poly-(D)-lysine	18,000

**Figure 8:**
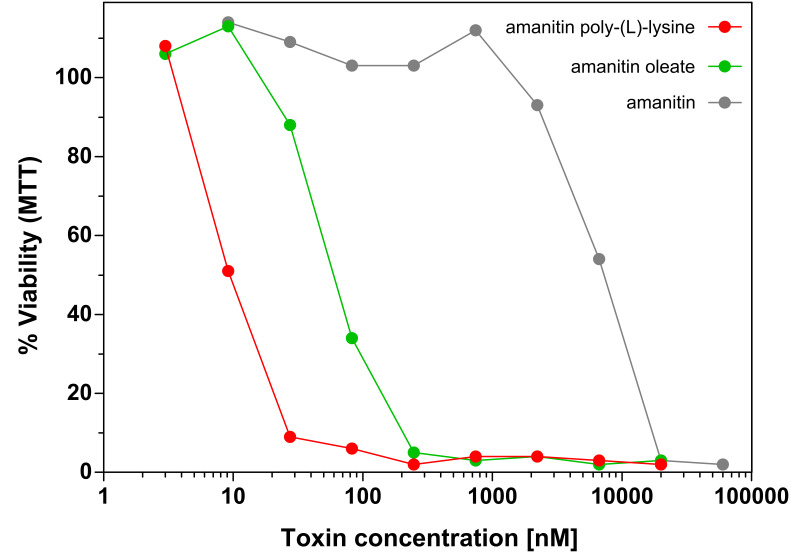
NIH 3T3 mouse fibroblasts were incubated with various concentrations of α-amanitin and amanitin derivatives. Cell viability was determined after 72 h incubation time by MTT assay.

Conjugation of amanitin to oleic acid increased the cytotoxicity by a factor of 150 (see Table 2). In analogy with the phallotoxins, we conclude that the oleic acid ester is hydrolysed inside the cell. β-Amanitin, when conjugated to poly-(L)-lysine, became 100-fold more toxic for mouse fibroblasts than the native toxin, showing IC_50_ values in the nanomolar range (Figure 8). As in the phallotoxin series, toxicity of β-amanitin conjugates depends on the cleavage of the toxin from the polymeric carrier, as shown by the fact that β-amanitin coupled to the (L)-polymer was 1800 times more toxic than when coupled to the (D)-polymer (Table 2).

## Discussion

### Growth inhibition as a parameter of internalization

Phallotoxins as well as amatoxins find their targets inside the cell. Thus, if growth inhibition by these toxins occurs the toxins must have penetrated the cell. Moreover, the extent of the toxic lesions will mirror the amount of toxin that penetrated the cell and, hence, can be used to estimate the amount of toxin taken up. Although some of the steps involved in the uptake process became evident in this study it was not our aim to investigate details of internalization, but to gather experience with moieties that may help to overcome the plasma membrane barrier and to shift hydrophilic peptides into a cell.

#### Previous experience with internalization-mediating moieties

Lipid acids have been used by various researchers to enhance the uptake of peptides and proteins into cells. For example Honeycutt et al. [14] used palmitic acid to deliver a protease inhibitor into cells, and Bradley et al. [15] used docosahexanoic acid to improve the uptake of paclitaxel into tumor cells. For a review see Wong and Toth [16]. Of particular interest was the incorporation of anti-sense oligonucleotides into cells by coupling them to lipophilic ligands as reported by Boutorin et al. [17], Letsinger et al. [18], and Shea et al. [19].

Polycationic carriers such as polylysine and polyarginine represent another, and possibly even more effective technique for delivering drugs into cells. Ryser and Shen [20] reported the internalization by polylysine of methothrexate and horse radish peroxidase; Leonetti et al. [21] the internalization of oligonucleotides; and Mulders et al. [22] the internalization of adenovirus into cells. A polylysine peptoid derivative was used by Murphy et al. [23] for “gene delivery”, and Emi et al. [24] used polyarginine as a carrier for gene transfer.

Protein transduction domains such as Tat-peptide, usually consisting of 10–30 amino acids, have been used to transport enzymes, drugs, liposomes and supermagnetic particles into cells [25–29]. In the meantime it has been shown that nona-arginine is many times more efficient than Tat-peptide, suggesting that the internalization effect of Tat-peptide is mainly due to its eight cationic side chains [30].

#### Attachment to plasma membrane determines the rate of internalization

Plasma membranes of cells in culture represent a lipophilic phase in an aqueous medium, and amphiphilic compounds brought into this system will distribute between the two phases according to their nature. This process is similar to the distribution the substance will take between *n*-octanol and water, an idea that has been suggested by Palm et al. [31].

Compounds **1a**–**1e** represent a series of phalloidin derivatives with increasing log *P*_ow_ values. At the same time they represent a series of phalloidin derivatives with increasing cytotoxic activity. Since the two parameters are linearly related (Figure 3), we conclude that the amount of toxin attached to the membrane determines the extent of the toxic effects observed. Clearly, this conclusion is valid only under the condition that, as in our case, all derivatives belong to the same chemical class, esters for example, and that processing inside the cell will yield the same toxic product, here native phalloidin, set free in all cases.

Attachment to the plasma membrane can also occur by electrostatic forces and, thus, may be behind the internalization effect observed with polylysine or oligoarginine as well. Plasma membranes expose numerous negatively charged components that can attract oligo- or polycationic molecules. As already pointed out, arginine residues as in **2d** are more effective than lysine residues in **2e**, as 120 lysine groups are required to match the internalization increase caused by eight arginine residues.

Concerning Tat-peptide, we believe that its eight cationic side chains interacting with the plasma membrane represent the more likely explanation for its internalization than other models proposed in the past.

Finally, tight attachment to the plasma membrane may also explain why the methoxy-polyethyleneglycol residue mediates internalization, since polyethyleneglycols are soluble not only in water but also in diethylether, i.e., can adopt a conformation capable of anchoring to the phospholipids in the plasma membrane. Lipophilic interaction of the aromatic part of tetramethylrhodamine with the plasma membrane is also thought to be the main cause of the internalization capacity of this fluorescent residue, beside its delocalized cationic charge on the two nitrogen atoms.

Although proved for a lipophilic phalloidin derivative only, we postulate that attachment to the plasma membrane also provides the first step in entering cells for the polycationic and the pegylated toxin derivatives presented in this study.

#### Internalization and processing

The uptake process following the binding step of the phalloidin derivative **3** to the plasma membrane was identified as endocytosis. As shown in Figure 6c, the fluorescent phalloidin is bound on the plasma membrane of mouse fibroblasts after 1 h, while after 6 h most of the fluorescent material resides in endocytotic vesicles. This finding confirms the earlier observation [6] that TRITC-phalloidin **3** enters isolated mouse hepatocytes by endocytosis and not through a phalloidin-transporting protein, such as the OATP1B1 present on human hepatocytes. The type of endocytosis seen here remains, however, to be elucidated. Likewise, we have no data on whether lipophilic or polycationic phallotoxins enter cells in the same way as the fluorescent toxin, but this seems likely since proteolytic enzymes present in endosomal-lysosomal compartments were found to be involved in the processing, and the difference in degradation after arrival in endosomes is the most likely explanation for the difference found in the toxicities of phalloidin when bound to either poly-(L)- or to poly-(D)-lysine (**2e**).

There is no evidence so far about how phalloidin released from the carrier finds its way out of the endosomal-lysosomal compartments into cytoplasm, where the target, the actin filaments, is located.

## Conclusions

With IC_50_ values of >10 mM (8 mg/mL; *M*_r_ 789 g/mol), phalloidin has so far been of no benefit for research on living cells, unless it was microinjected. This is regrettable, since the molecular mechanism of phalloidin action, the immobilization of the microfilament system, has been investigated in great detail. Moreover, phalloidin action is comparable to that of taxol, which induces comparable immobilization of the microtubular system and has been widely employed in cell research and even in tumor therapy [32–33]. One difference is that taxol is a lipophilic compound, and is thus active on cells at much lower concentrations. We balanced this disadvantage by coupling phalloidin, e.g., to oleic acid, allowing studies of the molecular toxicity of phalloidin on cells in the micromolar range and encouraging its use in tumor therapy.

The internalization-mediating effect observed with phalloidin was seen also with amatoxins. Although amanitin is active on cells in the micromolar range, it may be advantageous to use it bound to oleic acid or polylysine and thus decrease the medium concentration necessary to achieve growth inhibition down to the nanomolar range. Amanitin as a drug bound to tumor monoclonal antibodies, has recently been described for the therapy of adenocarcinomas [34].

Coupling to methoxypolyethyleneglycol has been described as a method to improve the solubility of drugs, prolong their half-lives in plasma, or modulate their pharmacokinetics [35]. An effect not described to our knowledge so far is that pegylation can enhance, several hundred-fold, the penetration of a hydrophilic drug into cells. Linkers with the drug should contain a disulfide bridge, as in compound (**2i**), to make sure that after internalization the drug is released in a defined and active form.

Likewise new, is the observation that the red fluorescent tetramethylrhodaminyl residue can facilitate internalization, e.g., of phalloidin, by a factor of >100. Linked by a thiourea moiety, as in compound **3**, the fluorescent moiety is not cleaved inside the cell, as concluded from the observation that this phalloidin derivative still decorates filaments in the cell. This property of rhodamine-labeled phalloidin seems useful as a tool for studying the kinetics of phalloidin-induced disturbances in the actin system of a cell in correlation with, for example, the development of apoptosis in the cell.

## Experimental

### Fatty acid esters of phalloidin

Ten micromoles of phalloidin was dissolved in 0.1 mL dry pyridine and reacted with 0.3 µmol of benzoyl chloride, salicyl chloride, octoyl chloride, myristoyl chloride, or oleoyl chloride for 2 h at rt. Under these conditions, the reaction proceeded predominantly at the primary OH group of the γ,δ-dihydroxyleucine residue in position 7 of phalloidin, with ca. 10–20% of esterification at the secondary OH groups. The reaction was stopped with 2 mL of methanol, and solvents were removed in vacuo at 60 °C. Purification of the esters was achieved by preparative TLC on silica (Merck HF_254_, Darmstadt; Germany) in chloroform/methanol/2 N acetic acid (65:25:4), followed by further purification of the methanolic extract of the silica on a Sephadex-LH20 column developed with methanol. Yields of the esters were 43% for benzoyl phalloidin, 35% for salicyl phalloidin, 46% for octoyl phalloidin, 50% for myristoyl phalloidin and 44% for oleoyl phalloidin; purities were 88 to 95% by HPLC.

#### Aminophalloidin

Ten micromoles of toluene-4-sulfonyl chloride in 2 mL chloroform were added to 0.6 µmol of phalloidin in 5 mL dry pyridine and allowed to react for 30 min on ice. The reaction was stopped by the addition of 50 mL of dry diethyl ether, and the sediment was isolated by centrifugation, washed twice with 50 mL diethyl ether, and dissolved in 5 mL methanol for separation on a Sephadex-LH20 (60 × 3 cm) column developed with methanol/H_2_O (1:1). Yield of the monotosylphalloidin was 65%; purity 94%. The vacuum-dried monotosylphalloidin was dissolved in 40 mL of methanol containing 2.5 N ammonia and reacted for 2 h. After evaporation in vacuo at 60 °C, the aminophalloidin was purified on a Sephadex-LH20 column with methanol as eluant. Yield of aminophalloidin was 80%, purity 89% by HPLC.

#### Fatty acid amides of phalloidin

Aminophalloidin and the fatty acid chlorides were reacted as described for the synthesis of phalloidin esters.

#### Linear peptides linked to aminophalloidin

Linear peptides such as Ac-Cys-Gly-Tyr-Gly-Arg-Lys-Lys-Arg-Arg-Gln-Arg-Arg-Arg-OH (Tat peptide), and Ac-Cys-Gly-Arg_8_-OH (Arg_8_) were synthesized on an Eppendorf Ecosyn P solid-phase synthesizer by using 9-fluorenylmethoxycarbonyl (Fmoc)-Arg(Pbf)SPHB resin (Rapp Polymere, Tübingen, Germany). For the Kaposi sequence Ac-Cys-Gly-Ala-Ala-Val-Ala-Leu-Leu-Pro-Ala-Val-Leu-Leu-Ala-Leu-Leu-Ala-Pro-OH an Fmoc-Pro-Trt-Tentagel resin was used. All amino acids were incorporated with the α-amino functions protected with the Fmoc group. Side chain functions were protected as *tert*-butyl ethers (tyrosine), *tert*-butyloxycarbonyl derivatives (lysine), trityl derivatives (cysteine, glutamine), and as (2,2,4,6,7-pentamethyl)dihydrobenzofuran-5-sulfonyl derivative (arginine). Coupling was performed by using a 4-fold excess of each of the protected amino acids and the coupling reagent 2-(1*H*-benzotriazole-1-yl)-1,1,3,3-tetramethyluronium tetrafluoborate (TBTU) and 2 equiv of diisopropylethylamine (DIEA) over the resin loading. Before the coupling steps the Fmoc groups were removed from the last amino acid of the growing peptide fragment by using 25% piperidine in dimethylformamide. After cleavage of the N-terminal Fmoc group, the peptide was removed from the resin under simultaneous cleavage of the amino-side-chain protecting groups by incubation for 3 h in a mixture of trifluoroacetic acid (TFA) (12 mL), ethanedithiol (0.6 mL), anisole (0.3 mL), water (0.3 mL), and triisopropylsilane (0.15 mL). The mixture was filtered, and washed with TFA and anhydrous diethyl ether. The crude products were further purified by HPLC on a Nucleosil 100 C18 (7 µm) column (250 × 10 mm, Macherey & Nagel, Düren, Germany) by using a gradient from 10–90% B in 32 min (solution A: 0.07% TFA/H_2_O; solution B: 0.059% TFA in 80% acetonitrile). The elution was monitored at 214 nm. The peptides were assayed for purity by analytical HPLC and ESIMS. The peptides were coupled to aminophalloidin through the hetero-bifunctional cross-linking agent SPDP (3-(2-pyridyldithio)propionic acid *N*-hydroxysuccinimide ester). Thus, the activated ester end of excess SPDP was reacted with the primary amine group in aminophalloidin to form an amide linkage. In general, 63 µmol aminophalloidin were dissolved in 3.4 mL H_2_O and 1.7 equiv SPDP, dissolved in 900 µL dimethylformamide, were added. The solution was adjusted to pH 7.5 with 1 N NaOH; reaction time was 1 h. Separation of the products was performed by Sephadex-LH20 column developed with methanol. The purity of PDP-aminophalloidin was 93%; the yield was about 65%. The 2-pyridyldithiol group at the other end of the linker was reacted with the sulfhydryl in the amino terminal Ac-Cys-Gly moiety of the linear peptides to form a disulfide group. The reaction conditions of the three linear peptides varied slightly and were as follows: 33 µmol Tat-peptide, dissolved in 1.0 mL PBS was added to 40 µmol of PDP-aminophalloidin dissolved in 0.5 mL methanol; 13.6 µmol Arg_8_-peptide, dissolved in 1.5 mL PBS was added to 28 µmol of PDP-aminophalloidin dissolved in 0.25 mL methanol; 12 µmol Kaposi-peptide, dissolved in 0.8 mL PBS was added to 36 µmol of PDP-aminophalloidin dissolved in 0.2 mL methanol. Reaction time was in all cases 16 h at rt. Separation was achieved on Sephadex-LH20 with H_2_O/methanol (4:1) as solvent. Yield for Tat-phalloidin was 52%, for Arg_8_-phalloidin 43% and for Kaposi-peptide 39%. Purity of all conjugates was >90% as shown by HPLC (Table 1) and MALDI–TOF analysis. The HPLC analysis conditions were as follows: Column Knauer RP Nucleosil-100 C18 (250 × 4 mm); mobile phase was a linear gradient of buffer A H_2_O, 0.05% TFA and buffer B acetonitrile/H_2_O (9:1), 0.05% TFA.; flow rate, 1.2 mL/min.

#### General

MALDI–TOF analysis was performed in the linear, high-mass, positive-ion mode with pulsed (time-delayed) extraction on a Kratos Maldi IV instrument (Shimadzu Deutschland, Duisburg, Germany). Samples (usually in 0.1% TFA) were either applied by a sandwich technique (in which 0.7 µL of matrix was dried onto the sample spot, followed by 0.7 µL of sample and then another 0.7 µL of matrix) or, for more concentrated samples, were simply mixed 1:10 with the matrix solution and 0.7 µL of this mixture was dried onto the stainless-steel sample holder. Matrix solutions were usually α-cyano-4-hydroxycinnamic acid [dissolved at 10 mg/mL in 50% acetonitrile, 50% 0.1% TFA (all % v/v)]. Spectra were calibrated by using near-external standards consisting of a mixture of fragment 1–4 of substance P ([M + H] *m*/*z* 497.6); angiotensin II ([M + H] *m*/*z* 1047.2); angiotensin I ([M + H] *m*/*z* 1297.5); fragment 1–13 of angiotensinogen ([M + H] *m*/*z* 1646.9); and oxidized insulin B chain ([M + H] *m*/*z* 3496.9). Absolute *m*/*z* values occasionally varied up to 1 Da depending on the individual calibration and the distance and time between the individual measurements; for these samples, spectra were recalibrated by using known *m*/*z* values of the largest peak(s). Spectra were collected and analyzed by using standard Kratos software (Sun OS, Release 5.4, OpenWindows Ver. 3.4, Kratos Kompact Software Ver. 5.2.0) and were usually the average of 50–100 individual laser shots across the width of the sample spot. Data were smoothed and baseline-corrected, generally with a window width of 30 channels.

#### Polymers linked to aminophalloidin

Poly-(L)-lysine (hydrobromide; *M*_r_ = 27500), and monomethoxy-polyethyleneglycolamine (*M*_r_ = 810, 5200, 22600) were coupled to aminophalloidin by the amine-reactive homo-bifunctional cross-linking reagents DSP (dithiobis(succinimidylpropionate); Lomant’s reagent) with cleavable disulfide group, or DSS (disuccinimidyl suberate) containing a hydrocarbon chain instead of the disulfide group. DSP or DSS (248 µmol) were dissolved in 1.0 mL of *N*,*N*-dimethylformamide and added to 63 µmol dried aminophalloidin. The reaction was started with 2 µL triethylamine and allowed to proceed under magnetic stirring for 16 h at rt. The reaction was stopped with 10 mL diethyl ether and the mixture was centrifuged; after a second wash with ether, the sediment was dissolved in 5 mL methanol and separated on a Sephadex-LH20 column with methanol as solvent. Yield of DSP-, and DSS-phalloidin was about 80%, purity >90% for both.

Seven milligrams DSP-, or DSS-phalloidin were dissolved in 0.5 mL *N*,*N*-dimethylformamide and 5 equiv of poly-(L)-lysine hydrobromide or poly-(D)-lysine hydrobromide added in 0.5 mL PBS. After reaction for 16 h at rt, high-molecular-weight products were separated by gel-filtration chromatography with Sephadex G-25 with 0.1% NaCl as eluant. After lyophilisation, the amount of phalloidin coupled to the polymer was determined from the characteristic absorption of phalloidin at 300 nm (ε = 10,100). We found that ca. 1 out of ca.10 lysine residues was spiked with aminophalloidin, independent of the molecular weight of the polymer. For modification of DSP-phalloidin and DSS-phalloidin with methoxypolyethyleneglycolamine, monomethoxypolyethyleneglycol was tosylated and reacted with ammonia to yield monomethoxy-PEG with a reactive amino group: 100 mg monomethoxy-PEG 810, 5,200 and 22,600 were dissolved in 1.0 mL of dry pyridine in a round-bottom flask on ice, and 5 equiv toluol-4-sulfonylchloride in 0.4 mL chloroform were added dropwise. After being stirred for 30 min, the reaction was stopped with 20 mL of diethyl ether. Sediment was dried in a rotation evaporator and reacted with 20 mL of methanol/2.5 N ammonia. After 1 h, the solvent was evaporated in vacuo and the aminomonomethoxy-PEG purified by Sephadex-LH20 chromatography. Ten milligrams DSP- or DSS-aminophalloidin were dissolved in 0.5 mL *N*,*N*-dimethylformamide and added to 1 equiv dry aminomonomethoxy-PEG. After reaction for 16 h at rt, phalloidin PEG 22,600 and phalloidin PEG 5,200 were purified on a Sephadex G25 column by using 0.1% NaCl as solvent, and phalloidin PEG 810 was purified on a Sephadex-LH20 column developed with methanol. The yield was 37% for phalloidin PEG 22,600, 34% for phalloidin PEG 5,200 and 45% for phalloidin PEG 810.

#### Affinity to rabbit muscle actin

Actin was prepared from rabbit muscle as described previously [36]. The binding assay was used with the following modifications: Freshly prepared G-actin solution was diluted in Tris-ATP buffer (2 mM of Tris; 0.2 mM ATP; 0.1 mM CaCl_2_; 0.02% NaN_3_; pH 7.8) to an extinction of 0.28 at 290 nm (null balance at 310 nm). Typically, 18 µL of a [^3^H]-demethylphalloin methanolic solution (specific activity 9 Ci/mmol) was evaporated in a scintillation tube by a hot-air dryer, and 1.5 mL of G-actin solution was added. Polymerisation was initiated by addition of 12.5 µL 0.2 M MgCl_2_. After 30 min incubation at room temperature the solution was filled up to ten volumes with Tris-ATP buffer and gently homogenized. Hydrophilic toxin derivatives were dissolved in potassium Tris-buffer (100 mM KCl, 1 mM Tris, pH 7.4). The concentration was determined spectrophotometrically at 300 nm (ε (300 nm) = 10100 M^−1^ cm^−1^). Hydrophobic toxin derivatives, because of low solubility in aqueous solvents, were dissolved in methanol. Concentration was determined spectophotometrically at 300 nm. The methanolic solution was filled up to three volumes with potassium Tris-buffer, and a dilution series established down to a concentration of 10^−7^ M. Fifty microliters of this solution was added to 450 µL of the above actin solution and allowed to equilibrate for 1 h. Samples of 180 µL of this solution were transferred to Ti 42.2 centrifugation tubes (Beckman) and centrifuged at 40.000 rpm for 50 min at 10 °C. Twenty microliter aliquots of the supernatant were counted, and affinity values based on phalloidin = 1 were calculated from those concentrations of phallotoxins required for a 50% substitution of the [^3^H]-demethylphalloin.

#### MTT proliferation assay

Mouse fibroblasts NIH 3T3 (generous donation from Prof. Traub, MPI Ladenburg), K562, HL-60 and Jurkat cells (ATCC) maintained in RPMI 1640 medium containing 10% fetal calf serum and 0.05 mM β-mercaptoethanol, were cultured at 37 °C in a humidified 95% air/5% CO_2_ incubator. The cytotoxicity of phallotoxins was assessed by using the 3-(4,5-dimethylthiazol-2-yl)-2,5-diphenyltetrazolium bromide (MTT) assay. Exponentially growing cells were plated at a density of 2 × 10^4^ cells/well in 96-well plates 24 h before the toxin was added in medium with up to 1% DMSO, with a volume equal to the volume of medium in the culture dish. The final concentrations of toxins in the media were between 10^−3^ and 10^−9^ M. At 72 h, the medium was replaced by serum-free medium containing 25 µL MTT solution (5 mg/mL in PBS), and the incubation was continued at 37 °C for 4 h. Then, lysis buffer (100 µL 20% SDS in 50% dimethylformamide) was added to each well and incubated for another 16–20 h. Viability of cells was determined by measuring the 570 nm absorbance of each well using a microplate reader (Molecular Devices). IC_50_ values were calculated as the concentration of toxin required to reduce the absorbance to 50% of the control cultures.

#### Microscopic studies

Exponentially growing fibroblasts were plated at a density of 2 × 104 cells/well in glass-bottom dishes 24 h before the fluorescently labeled peptides were added. The final concentration of the peptides was 10^−5^ M. After different incubation times the cells were washed with fresh medium, and microscopic studies were performed by using a confocal laser scanning microscope TCS SP2 (Leica Microsystems, Heidelberg/Mannheim, Germany), equipped with an inverted microscope DMIRE2 and an incubation chamber (Pe-Con Erbach, Germany). Image data stacks and time-lapse studies of the live cells were obtained at 37 °C and in 5% CO_2_ atmosphere with a 100×/1.4 N.A. oil immersion objective and CLSM software (Leica Microsystems). The data were processed with ImageJ software, optimizing images in brightness and contrast, and visualization of the fluorescing structures was performed on serial confocal optical sections.

## Supporting Information

File 1 Structures of phalloidin derivatives and IC_50_ concentration values of cell growth inhibition.

## References

[1] Wieland T (1986). Peptides of Poisonous Amanita Mushrooms.

[2] Roeder R G, Rutter W J (1969). Nature.

[3] Chambon P, Gissinger F, Kedinger C (1974). The Cell Nucleus.

[4] Wehland J, Weber K (1981). Eur J Cell Biol.

[5] Wulf E, Deboben A, Bautz F A, Faulstich H, Wieland T (1979). Proc Natl Acad Sci U S A.

[6] Faulstich H, Trischmann H, Mayer D (1983). Exp Cell Res.

[7] Bushnell D A, Cramer P, Kornberg R D (2002). Proc Natl Acad Sci U S A.

[8] Fehrenbach T, Cui Y, Faulstich H, Keppler D (2003). Naunyn Schmiedebergs Arch Pharmacol.

[9] Meier-Abt F, Faulstich H, Hagenbuch B (2004). Biochim Biophys Acta.

[10] Letschert K, Faulstich H, Keller D, Keppler D (2006). Toxicol Sci.

[11] Heitz F, Morris M C, Divita G (2009). Br J Pharmacol.

[12] Jones A T, Sayers E J (2012). J Control Release.

[R13] 13Anderl, J. *Synthesis and cytotoxicity of membrane-permeable phallotoxins*, Doctoral Thesis, Heidelberg University, Germany, 2003.

[14] Honeycutt L, Wang J, Ekrami H, Shen W-C (1996). Pharm Res.

[15] Bradley M O, Swindell C S, Anthony F H, Witman P A, Devanesan P, Webb N L, Baker S D, Wolff A C, Donehower R C (2001). J Control Release.

[16] Wong A, Toth I (2001). Curr Med Chem.

[17] Boutorin A S, Gus'kova L V, Ivanova E M, Kobetz N D, Zarytova V F, Ryte A S, Yurchenko L V, Vlassov V V (1989). FEBS Lett.

[18] Letsinger R L, Zhang G R, Sun D K, Ikeuchi T, Sarin P S (1989). Proc Natl Acad Sci U S A.

[19] Shea R G, Masters J C, Bischofberger N (1990). Nucleic Acids Res.

[20] Ryser H J-P, Shen W-C (1978). Proc Natl Acad Sci U S A.

[21] Leonetti J P, Degols G, Lebleu B (1990). Bioconj Chem.

[22] Mulders P, Pang S, Dannull J, Kaboo R, Hinkel A, Michel K, Tso C-L, Roth M, Belldegrun A (1998). Cancer Res.

[23] Murphy J E, Uno T, Hamer J D, Cohen F E, Dwarki V, Zuckermann R N (1998). Proc Natl Acad Sci U S A.

[24] Emi N, Kidoaki S, Yoshikawa K, Saito H (1997). Biochem Biophys Res Commun.

[25] Fawell S, Seery J, Daikh Y, Moore C, Chen L L, Pepinsky B, Barsoum J (1994). Proc Natl Acad Sci U S A.

[26] Anderson D C, Nichols E, Manger R, Woodle D, Barry M, Fritzberg A R (1993). Biochem Biophys Res Commun.

[27] Allinquant B, Hantraye P, Mailleux P, Moya K, Bouillot C, Prochiantz A (1995). J Cell Biol.

[28] Rothbard J B, Garlington S, Lin Q, Kirschberg T, Kreider E, McGrane P L, Wender P A, Khavari P A (2000). Nat Med.

[29] Torchilin V P, Rammohan R, Weissig V, Levchenko T S (2001). Proc Natl Acad Sci U S A.

[30] Wender P A, Mitchell D J, Pattabiraman K, Pelkey E T, Steinman L, Rothbard J B (2000). Proc Natl Acad Sci U S A.

[31] Palm K, Luthman K, Ungell A-L, Strandlund G, Artursson P (1996). J Pharm Sci.

[32] Schiff P B, Horwitz S B (1980). Proc Natl Acad Sci U S A.

[33] Perez E A (2009). Mol Cancer Ther.

[34] Moldenhauer G, Salnikov A V, Lüttgau S, Herr I, Anderl J, Faulstich H (2012). J Natl Cancer Inst.

[35] Veronese F M, Mero A (2008). BioDrugs.

[36] Spudich J A, Watt S (1971). J Biol Chem.

