# Supplementary material for: Chemical modification allows phallotoxins and amatoxins to be used as tools in cell biology
Source: Beilstein J Org Chem. 2012 Nov 27;8:2072–84. doi: 10.3762/bjoc.8.233 (PMC3511042; doi:10.3762/bjoc.8.233)
Supplement: File 1 — Structures of phalloidin derivatives and IC50 concentration values of cell growth inhibition. [file Beilstein_J_Org_Chem-08-2072-s001.pdf]

Supporting Information

for

Chemical modification allows phallotoxins and amatoxins to be used as tools in cell biology

Jan Anderl[1], Hartmut Echner[2] and Heinz Faulstich*[3]

Address: [1]Heidelberg Pharma GmbH, Schriesheimer Str. 101, 68526, Ladenburg, Germany, [2]University of Tübingen, Medical School, Hoppe-Seyler-Str. 3, 72076, Tübingen, Germany, and [3]Max-Planck Institute for Medical Research, Jahnstr. 29, 69120, Heidelberg, Germany

Email: Heinz Faulstich - hfaulstich@web.de

* Corresponding author

Structures of phalloidin derivatives and IC_{50} concentration values of cell growth inhibition

Table S1: Structures of phalloidin derivatives and IC_{50} values of cell growth inhibition after 72 h incubation time determined by MTT cell proliferation assay (n.a.: not assayed).

	R	Name	IC_{50} values K562 cells [µM]	IC_{50} values HL-60 cells [µM]	IC_{50} values Daudi cells [µM]
**1**	OH	phalloidin	>1,000	>1,000	>1,000
**1a**	$OCOC_6H_5$	(**1**)-benzoate	26	90	70
**1b**	$OCOC_6H_4OH$	(**1**)-salicylate	28	83	100
**1c**	$OCOC_7H_{15}$	(**1**)-octanoate	n.a.	n.a.	n.a.
**1d**	$OCOC_{13}H_{27}$	(**1**)-myristate	n.a.	n.a.	n.a.
**1e**	$OCO(CH_2)_7CHCH(CH_2)_7CH_3$	(**1**)-oleate	2	3	4
**2**	NH_2	aminophalloidin	>1,000	>1,000	>1,000
**2a**	$NHCOC_6H_5$	*N*-benzoyl-(**2**)	26	90	70
**2b**	$NHCO(CH_2)_7CHCH(CH_2)_7CH_3$	*N*-oleoyl-(**2**)	n.a.	n.a.	n.a.
**2c**	$NHCO(CH_2)_2SS(Ac)CysGlyTyrGlyArg$- -$(Lys)_2(Arg)_2Glu(Arg)_3OH$	(**2**)-Tat-peptide	4	6	4
**2d**	$NHCO(CH_2)_2SS(Ac)CysGly(Arg)_8OH$	(**2**)-octarginine	n.a.	n.a.	n.a.
**2e**	$NHCO(CH_2)_6CONH(Lys)_{210}$	(**2**)-poly-(L)-$lysine_{28,000}$	n.a.	n.a.	n.a.
		(**2**)-poly-(D)-$lysine_{28,000}$	n.a.	n.a.	n.a.
**2f**	$NHCO(CH_2)_2SS(CH_2)_2CONH(Lys)_{210}$	(**2**)-(SS) poly-(L)-$lysine_{28,000}$	4	2	2
		(**2**)-(SS) poly-(D)-$lysine_{28,000}$	n.a.	n.a.	n.a.
**2g**	$NH(CH_2)_2SS(CH_2)_2CONH(PEG)_{800}$	(**2**)-(SS) PEG_{800}	n.a.	n.a.	n.a.
**2h**	$NH(CH_2)_2SS(CH_2)_2CONH(PEG)_{5,200}$	(**2**)-(SS) $PEG_{5,.200}$	64	93	84
**2i**	$NH(CH_2)_2SS(CH_2)_2CONH(PEG)_{23,000}$	(**2**)-(SS) $PEG_{23,000}$	n.a.	n.a.	n.a.
**2f +DTT**	$NH(CH_2)_2SH$	*N*-(2-mercaptoethyl)-(**2**)-SH	n.a.	n.a.	n.a.
**3**		dithiolanoaminophalloidin TRITC labeled	n.a.	n.a.	n.a
